# Plant genome and transcriptome annotations: from misconceptions to simple solutions

**DOI:** 10.1093/bib/bbw135

**Published:** 2017-01-05

**Authors:** Marie E Bolger, Borjana Arsova, Björn Usadel

**Affiliations:** 1 Forschungszentrum Jülich, Wilhelm Johnen Str, Jülich, Germany; 2 FRS-FNRS Chargé de Recherches, Functional Genomics and Plant Molecular Imaging Center for Protein Engineering (CIP), Dpt of Life Sciences, University of Liège, Quartier de la Vallée, 1, Chemin de la Vallée, 4 - Bât B22, 4000 LIEGE, Belgium; 3 RWTH Aachen University, Institute for Biology I Botany, BioSC, Worringer Weg 3, Aachen, Germany

**Keywords:** plant genome annotation, plant ontologies, plant gene family databases, genome annotation pipelines

## Abstract

Next-generation sequencing has triggered an explosion of available genomic and transcriptomic resources in the plant sciences. Although genome and transcriptome sequencing has become orders of magnitudes cheaper and more efficient, often the functional annotation process is lagging behind. This might be hampered by the lack of a comprehensive enumeration of simple-to-use tools available to the plant researcher. In this comprehensive review, we present (i) typical ontologies to be used in the plant sciences, (ii) useful databases and resources used for functional annotation, (iii) what to expect from an annotated plant genome, (iv) an automated annotation pipeline and (v) a recipe and reference chart outlining typical steps used to annotate plant genomes/transcriptomes using publicly available resources.

## Introduction

Next-generation sequencing has triggered an explosion of available genomic and transcriptomic resources in the plant sciences [1]. Since the genome sequence of the model plant *Arabidopsis thaliana* was published in 2000 [2], around 180 plant genome sequences have been published (http://www.plabipd.de/portal/sequence-timeline, https://en.wikipedia.org/wiki/List_of_sequenced_plant_genomes). This number is greatly enhanced by including plant transcriptome assemblies. As of August 2016, the transcriptome shotgun assembly database of the National Center for Biotechnology Information (NCBI) lists over 450 plant assemblies (https://www.ncbi.nlm.nih.gov/Traces/wgs/?view=TSA), whereas the plant 1KP project alone (onekp.com) includes >1300 plant transcriptomes. This is further complemented by countless plant transcriptomes found in Supplemental Materials. This remarkable surge is a testament to the genomics revolution that has provided us with the tools to quickly sequence whole transcriptomes on a relatively modest budget, which typically can yield sufficient data for a working quality transcriptomic inventory (‘the transcriptome’).

Generating an assembly for a species is merely the first step in the elucidation of the genome. Extensive processing and analysis is necessary before the resource will yield scientific insights. In the case of genome assemblies, a process called structural annotation is necessary. This process detects genes including their exon/intron structures within a given assembly. Although this can rely on extensive ‘extrinsic evidence’ in the form of RNA sequence (RNASeq) [3], this is often complemented by sophisticated statistical models of gene structures to find exon/intron structures in what is termed *ab initio* discovery. This process has been covered in detail, and readers are referred to [4]. Current popular tools to structurally annotate a genome include the automated pipelines MAKER-P specifically developed for plants [5] and the generalist BRAKER1 [6].

Assembling RNASeq data to produce high-quality transcriptome assemblies as a shortcut to a ‘functional genome’ [7] is still not a trivial task, despite these data sets being typically smaller and consisting uniquely of gene rich data. Popular transcriptome assembly tools such as TRINITY [8] require significant optimization to produce an assembly of reasonable quality. For recipes and cookbooks in the plant field one can refer to [9–11].

Once the gene structures have been detected, the necessary next step is to ascribe biological function to the genes in a process known as functional annotation. Surprisingly, performing this task to a degree of accuracy remains challenging, despite the extensive accrual of knowledge about gene function in model and crop species. Indeed, there is still a large percentage of genes, many of which can be found across multiple species, whose function has not been ascertained.

Within the plant community, *A. thaliana* remains the best annotated plant largely because of the tremendous effort of The Arabidopsis Information Resource (TAIR), which integrates community-based curations together with annotations from literature evidence. Over 2800 experimentally supported annotations have been included within the past 2 years alone [12]. This wealth of data has been adopted and further augmented by AraPort [13], an open-source resource, which encourages the community to contribute not only data modules but visualization tools and apps. Despite these extensive resources, published data [14] indicate that still only about 77% of the protein-coding sequences could be assigned any kind of structured annotation. This figure is in agreement with data from the PLAZA database, an online platform that has processed the annotations from several plant species into a uniform format [15].

## Controlled terms and vocabularies for plant functional annotations

Homology-based functional annotation is the transfer of existing knowledge about a gene sequence to another gene sequence within the same species or to another species. This process essentially depends on the existing knowledge about a gene function being transferable to genes of a similar sequence and assumes that this similarity reflects functional homology. Although an experimentalist working with a non-model species may likely be content with an annotation such as ‘quite similar to an *Arabidopsis thaliana* malic enzyme’, this annotation bears several difficulties for a sustainable annotation framework. This also hampers structured analysis of genome-wide data to answer questions like, ‘how many genes are involved in photosynthesis or glycolysis?’. This problem can largely be alleviated by using controlled vocabularies and functional ontologies [16] to provide a consistent description of gene products across different species.

### The Gene Ontology ontology

The most widely used functional annotation is ‘Gene Ontology’ (GO) that provides defined ‘GO terms’ to enable gene products to be described by three separate domains: ‘Biological Process’, which describes the gene in terms of a recognized series of events or molecular functions, ‘Cellular Component’ describing the location of a protein (or rather biomolecule) at a cellular and/or macromolecular level and ‘Molecular Function’ describing the jobs or abilities that a gene product has on the molecular level. Besides GO terms, each GO annotation contains an ‘evidence code’, which provides information on how a GO term was applied to a gene. Evidence codes indicate whether the annotation is based on experimental evidence, computational analysis, author statements or curatorial statements, all of which are manually curated. GO annotations also contain evidence codes, which are used to indicate assignment by automatic/computational methods. This has the advantage that annotations based on experimental data can be treated with much higher confidence than automatic annotations of related proteins. In addition, by qualifying where such an annotation came from it is easier to check the respective annotation. In this respect, curated GO resources such as the one for *A. thaliana* represent an invaluable resource.

The GO ontology is structured as a directed acyclical graph making it possible to infer more general terms from a specific term. This additionally allows grouping data, e.g. our malic enzyme might be annotated with the GO term ‘GO:0009763’ ‘NAD-malic enzyme C4 photosynthesis’ from which one could immediately deduce using, e.g., the Amigo browser [17] that the terms ‘GO:0015979’ ‘photosynthesis’ and ‘GO:0015977’ ‘carbon fixation’ also apply.

### The Kyoto Encyclopedia of Genes and Genomes ontology

Another widely used resource is the Kyoto Encyclopedia of Genes and Genomes (KEGG http://www.kegg.jp/). This features a number of databases that aim to link genomic- and molecular-level information to higher-level functions of the cell, organism and the ecosystem. Annotation with KEGG is based on associating molecular function with orthologous groups, which are defined based on clustering of genes from completed genomes (currently, >4000 genomes), using the KEGG’s internal ‘KEGG Orthology and Links Annotation’ (KOALA) program. The resulting information is stored in the ‘KEGG Orthology’ (KO) database, and assignment of KO entry identifier (also called K numbers) provides the gene annotation. KEGG aims to include reference to primary literature for each KO entry (76% of around 19 000 KO entries contains this as of September 2015) [18].

### CYC and other metabolic resources

In the case of enzymatic reactions, there is also the CYC network, whose plant section is under the Plant Metabolic network (http://www.plantcyc.org) umbrella [19]. This is mainly used to describe enzymatic functions, and while it enables one to build reaction networks [20], it does not cover additional functional terms. The plant Reactome is a database of plant metabolic and regulatory pathways which have been curated for the reference species rice and applied to 58 other plant species [21]. Finally, the Enzyme Commission numbers [22] (http://www.chem.qmul.ac.uk/iubmb/enzyme) describe reactions and classify enzymes, which are also referenced by KEGG, CYC and Reactome. Although CYC uses citations to the primary literature extensively, the enzymes within CYC are not formally linked to annotations via evidence codes.

### The MapMan BIN ontology

One additional ontology resource that is specifically plant focused is the MapMan BIN ontology. This was originally devised to visualize omics data on plant pathways [23] but has grown since and currently comprises around 2000 ontological terms. The MapMan ontology is modeled in a hierarchical tree structure with higher-level categories based on biological process and leaf categories containing detailed function. The structure was manually defined by experts in the respective fields, and changes are applied periodically based on primary literature. Although MapMan endeavors to assign ‘evidence’ to the BINs (http://mapman.gabipd.org/web/guest/mapcave), these are currently updated as new releases are published. As this ontology is strictly plant-specific, it lacks non-plant terms featured by, e.g., GO, KO and the CYC databases.

## Common misconceptions

Functional annotation usually depends on the transfer of functional knowledge from one gene to another. This assumes that the initial functional annotation is not only correct but also of a ‘robust nature’ to allow transfer. There are many pitfalls, which can occur during this transfer process, and which may, ultimately, lead to either incomplete or missannotation of genes.

### The ‘annotatable’ gene space in plants

**Figure 1.**
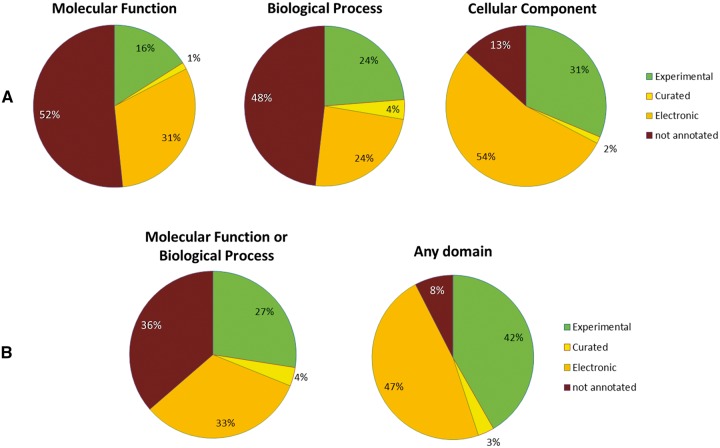
Overview of the number of annotated genes for the genome of the model plant *A. thaliana* based on analysis of GO terms. The GoSlim annotations were downloaded from the TAIR Web site (ftp://ftp.arabidopsis.org/Ontologies/Gene_Ontology/ATH_GO_GOSLIM.txt—downloaded July 2016). For each of the three main GO domains, the respective annotations were categorized according to the evidence code. The ‘Experimental’ category includes genes annotated with evidence codes IDA (inferred from direct assay), IMP (inferred from mutant phenotype), IGI (inferred from genetic interaction), IPI (inferred from physical interaction) or IEP (inferred from expression profile). ‘Curated’ includes those which had evidence codes IC (Inferred by Curator), NAS (Non-traceable Author Statement) and TAS (Traceable Author Statement) but lacking any annotation covered by the ‘Experimental’ category. ‘Electronic’ includes genes annotated with evidence codes ISS (Inferred from Sequence or Structural Similarity), ISO (Inferred from Sequence Orthology), ISM (Inferred from Sequence Model), IBA (Inferred from Biological Aspect of Ancestor), RCA (Inferred from Reviewed Computational Analysis) or IEA (Inferred from Electronic Annotation), but lacking any annotation from the ‘Experimental’ or ‘Curated’ categories. (**A**) The three aspects are shown separately. (**B**) The best annotation from multiple domains is shown, with the combination of Molecular Function and Biological Process on the left, and all three domains combined on the right.

The number of annotated genes in an assembly is a frequently used assessment in published data [24]. Before one can assess the results of this, one needs to first know how many genes can be annotated. This question is far from easy to answer, as it varies not only between species but also varies depending on what is considered as a ‘high confidence’ annotation. The use of the ontologies mentioned previously highlights that the function of many genes remain ‘dark matter’. The data shown in Figure 1 give an upper bound based on the best annotated *A. thaliana* genome. When one considers annotations pertaining to a molecular function or biological process separately, slightly >50% of the *A. thaliana* genes can be assigned a GO function (Figure 1A). Even when asking whether a gene has a molecular function or a biological process annotated to it, in our test data, the number reached 64% (Figure 1B). The numbers are naturally lower when one only considers experimental evidence data and not electronic annotations, which are often based on homology transfer. Only in the case of ‘cellular component’ are these numbers much higher (Figure 1A), as the subcellular localization can usually be predicted easily as shown in the section ‘Subcellular localization’ below.

Put in other words, this means that obtaining functional annotations (based on Molecular function and Biological process, Figure 1B) for more than two-thirds of the plant protein-coding genes analyzed is relatively unlikely, and a number much lower than this could suggest an incomplete genome or transcriptome.

### Annotation quality can vary

Even in cases when genes have been successfully annotated, the question about the quality of the annotations needs to be addressed. One simple pitfall is to take sequence similarity to annotated proteins at face value. Indeed, any functional annotation derived by simple sequence similarity transfer should be scrutinized carefully before embarking on a particular hypothesis about this particular protein. Given that proteins generally consist of one or more distinct domains embedded in generic regions, annotations that only look at sequence similarity, but do not take into account that certain domains are necessary to exert a function, might lead to an incorrect annotation.

### Absence of annotation does not mean absence of function

Furthermore, absence of a specific annotated gene in a plant genome/transcriptome does not necessarily mean that the plant cannot perform a particular function. Functional annotation is highly dependent on complete gene models, so in cases of partial or incomplete gene models, as is frequently seen with transcriptome assemblies, the tools used might not be sensitive enough to ascribe (the correct) function on a partially assembled gene. Thus, caution needs to be exercised when posing hypotheses based on gene or even pathway loss. Such scenarios need to be carefully validated using manual approaches. A first step would be to analyze the genome/transcriptome specifically for this function by using, e.g., BLAST [25] or searching for a necessary domain using, e.g., HMMER3 [26] using the resources listed in Table 1. In the case of no good candidates, more sophisticated and even experimental methods would need to be used to demonstrate the absence of a gene function beyond reasonable doubt.

**Table 1.** Available resources for protein family- or domain-based functional identifications

Resource	Version	Families	Web address	Comments
PFAM	30.0	16 306	http://pfam.xfam.org/	
TIGRFAM	15.0	4488	http://www.jcvi.org/cgi-bin/ tigrfams/index.cgi	
PANTHER	11.0	13 096	http://pantherdb.org	
SMART	7.1	1312	http://smart.embl-heidelberg.de/	License necessary
EggNOG	4.5	190 648 (37 127 plants)	http://eggnogdb.embl.de/#/app/home	
INTERPROSCAN	58.0	>40 000 integrated entries	https://www.ebi.ac.uk/ interpro/search/sequence-search	Meta engine including all other resources except EggNOG but not necessarily the most recent version at all times
CDD	3.15	52 411 (11 474 from CDD curation)	http://www.ncbi.nlm.nih.gov/cdd/	Uses RPS-BLAST and includes partly older versions of PFAM, SMART and TIGRFAM

In conclusion, one should keep in mind that functional annotations should be treated with care and taken as working hypotheses that might or might not need to be verified by biological experimentation.

## Functional annotations using generic tools and *ad hoc* pipelines

Given the current levels of plant genome annotation, it is perhaps unsurprising that frequently, the sole annotation process used is based on sequence similarity to the well-annotated plant *A. thaliana*. Indeed, often a simple BLAST search is performed using the genome/transcriptome as a query and the *A. thaliana* proteome as a subject. This is because of the well-maintained and annotated *A. thaliana* genome. In addition to *A. thaliana*, a selection of plant protein reference files can be obtained from Phytozome [27] and/or Ensembl Plants [28], with manually curated data sets for all species available from UniProtKB/Swiss-Prot [29].

Many functional annotation tools require that the input data are protein sequences, and some tools, which can accept either nucleotide or protein sequence, show superior results when protein sequences are submitted. Thus, extracting high-quality protein sequences is often the first step in functional annotation. The genome structural annotation pipeline from AUGUSTUS/BRAKER1 [3] provides auxiliary scripts (http://augustus.gobics.de/binaries/scripts/), which will conveniently output the protein sequences after genome annotation into a FASTA file.

### Finding coding regions in transcriptome assemblies

*De novo* transcriptome assemblies, however, pose additional challenges, as coding sequences need to be identified and frameshift mutations corrected before protein conversion. ESTScan [30, 31], a program which can detect coding sequences in DNA, has been developed to perform this task but needs to be trained with examples before it is used on a specific data set. This program exploits bias in nucleotide usage found in coding sequences relative to noncoding sequences. Other heuristics such as identifying the longest open reading frame (ORF) or by searching for frames that code for functional domains using TransDecoder (https://github.com/TransDecoder) [32] present alternative approaches. FrameDP [33] and GenemarkS-T [34] perform a similar function, but use sophisticated methods, which remove the need for the training steps. FrameDP was developed to discover coding sequences in transcripts or transcript fragments, such as ESTs and is part of the TRAPID [35] integrated tool (discussed further below). GenmarkS-T provides an algorithm, which is somewhat robust against assembly errors, and has been shown to compare favorably with other existing tools. Despite showing superior performance when tested by the authors against Transdecoder and ESTScan, the authors noted problems arising when RNASeq-based assemblies gave rise to the transcript models. This is because the underlying transcript models contained multiple errors leading to concomitant problems in coding region finding [34].

Sequencing errors carried over from the assembly to the annotation process might create artificial amino acid mutations or insert stop codons in ORFs, shortening existing or creating non-expressed peptides. Proteomics experiments are vital in experimentally validating gene models originating from transcriptome assemblies by comparing the expressed/measured peptides with the *in silico* database, as described in [36, 37]. However, functional annotation can frequently deal with an inaccurate ORF as long as most of the true coding region is retained. This is because similarities can still be identified based on slightly truncated regions.

### Annotation based on profile hidden Markov models

Tools that specialize in identifying domains within a sequence have advantages over simple similarity comparisons, as domain sequences typically are highly conserved between genes. Domains are frequently represented as profile hidden Markov models (HMMs), which are deduced from multiple sequence alignments stemming from several species, thus capturing typical sequence diversity at individual residues. This provides a more sensitive way to approach the sequence annotation problem. Table 1 provides a list of the main tools, which use protein family models often in the form of profile HMMs. PFAM [38] is likely the best known resource in this area and can currently identify >16 000 families. TIGRFAM [39] is a manually curated resource, which provides HMMs for full-length proteins and shorter regions. PANTHER’s [40] distinguishing feature is that it splits families into subfamilies allowing for a fine-grained annotation. SMART focuses on regulatory domains, which are often more difficult to tackle [41]. Finally, the EggNOG database [42] provides access to precomputed orthologous groups including plant-specific ones, along with functional annotations.

Even though these resources do not necessarily attribute a specific function to a protein, they do provide valuable evidence or hints toward the function of the protein. In addition to these standalone resources, HMMs used by many of these tools can be downloaded (in some cases, after having applied for a license) and used with the HMMER software suite [26].

### The integrated InterProScan resource

Many of the protein family databases mentioned in the previous section contain overlapping information (e.g. the NAD-binding domain of a malic enzyme would be identified both by the PFAM HMM PF03949 and the SMART HMM SM00919). Thus, it is often beneficial to use InterProScan [43], as this platform brings such ‘redundant’ information from the different protein families under one common umbrella (for the malic enzyme NAD-binding domain regardless of whether it was identified via SMART, PFAM or both, it would assign the InterPro Identifier IPR012302 ‘Malic enzyme, NAD-binding’). InterProScan additionally can assign GO terms by mapping from InterPro identifiers to GO term(s) using a cross-mapper called Interpro2GO [44]. Even though InterProScan does not always support the latest version of all the databases, a single tool that offers a diverse range of databases is of great benefit to users. A notable non-HMM-based reference database offered by InterProScan is the Conserved Domain Database (CDD) [45], which like PFAM comprises protein domains, but also features full length protein alignments. CDD relies on RPS-BLAST and, thus, ultimately on position-specific scoring matrices [46] to identify sequence similarity. From a user’s perspective, it is interesting to note that CDD also incorporates data from PFAM, TIGRFAM and SMART offering another tool that incorporates several sources such as InterProScan. CDD offers the advantage of a simpler setup scenario than InterProScan, as it is based on RPS-BLAST.

### Using genome-scale orthology finding

To increase or improve functional annotations, genome-scale draft-quality orthology detection is frequently incorporated. This also helps in exploring protein family relationships and comparative genomic approaches. In the simplest case, this could be a reciprocal best BLAST hit, which offers a quick and easy way to obtain a one-to-one relationship table. Tools such as Inparanoid [47], Orthofinder [48] and OrthoMCL [49] use BLAST and clustering algorithms in a convenient pipeline. Each offers different benefits, and the performance of several tools has recently been compared by Altenhoff *et al*. [50]. However, it should be noted that incomplete transcriptomes/genomes can lead to misdetection of orthologs, as the proper ortholog might be missing in the incomplete transcriptome/genome. Also, especially for reconstructed transcriptomes, it is not possible to generate full-length sequences for all contigs. This leads to additional decreases in ortholog detection accuracy, which need to be accounted for.

In the case of closely related species, one can refine orthology prediction further if full-genome information is available by making use of synteny, i.e. that gene order remains conserved across species [51]. The online tool CoGe [52] offers an automated pipeline to perform this task. However, this is a specialized step that lies downstream of typical functional annotation.

## Adding information

In addition to gene function (captured by ‘molecular function’ or ‘biological process’ in the GO ontology), it can be useful to gain an insight into the topological considerations for plant proteins as well as their subcellular localization and potential posttranscriptional modification.

### Transmembrane domains

One approach for adding protein topology is predicting transmembrane domains based on the protein sequence. TMHMM [53] offers a simple Web-based solution for alpha helix detection and can also be downloaded as a standalone tool for academic use. The free tool TOPCONS [54], which is actively being developed, combines a selection of prediction tools to provide a consensus result. This has demonstrated better performance, but its local installation is slightly more complex than TMHMM because of software dependencies. A comprehensive listing of transmembrane domain prediction tools is available in the Aramemnon transmembrane database [55] and in a recent review [56] (Table 2).

**Table 2.** Available resources to complement functional annotation

Resource	Web address	Comments
TMHMM	http://www.cbs.dtu.dk/services/TMHMM/	Can be downloaded and installed locally for academics. Online version allows the submission of 10 000 sequences at most
TOPCONS	http://topcons.net/	Can be downloaded and installed freely (GPL v2). Online version allows the submission of 100 MB sequence data at most
TargetP	http://www.cbs.dtu.dk/services/TargetP/	Can be downloaded and installed locally for academics. The online version allows the submission of 2000 sequences at most
Plant-mPLoc	http://www.csbio.sjtu.edu.cn/bioinf/plant-multi/	At time of writing problem with multifasta submission
AtSubP	http://bioinfo3.noble.org/AtSubP/	Up to 2000 predictions
Predotar	https://urgi.versailles.inra.fr/predotar/predotar.html	Only N-terminal signals for mitochondria and chloroplasts
PHOSFER	http://saphire.usask.ca/saphire/phosfer/index.html	Free for academic use only
PhosPhAt	http://phosphat.uni-hohenheim.de/phosphat.html	
PlantPhos	http://csb.cse.yzu.edu.tw/PlantPhos/Predict.html	Uploads <2 MB
Musite	http://musite.net/	≤100 predictions; can be downloaded and installed locally freely (GPL v3)
TAIR/Protein Interaction Data	https://www.arabidopsis.org/download/index-auto.jsp?dir=%2Fdownload_files%2FProteins%2FProtein_interaction_data	
Arabidopsis Predicted Interactome and Arabidopsis interactions Viewer	ftp://ftp.arabidopsis.org/home/tair/Proteins/Protein_interaction_data/Interactome2.0/ or http://bar.utoronto.ca/interactions/ cgi-bin/arabidopsis_interactions_ viewer.cgi	Downloadable from TAIR, these are the data for interactome v2.0 (also available at the Arabidopsis Interactions viewer). In total, 70 000 predicted interactions and 3000 experimentally determined interactions
IntAct	http://www.ebi.ac.uk/intact/	Interactions from literature curations or user submissions; part of the IMEx consortium
AtPIN	http://atpin.bioinfoguy.net/cgi-bin/atpin.pl	Incorporates data from: IntAct, BioGRID, TAIR, Predicted Interactome for *Arabidopsis, AtPID*
ANAP	http://gmdd.shgmo.org/Computational-Biology/ANAP	Integrates 11 interaction databases
M.I.N.D	https://associomics.dpb.carnegiescience.edu/Associomics/Home.html	In total, 12 102 high-confidence protein–protein interactions, based on split-uniquitin system in yeast; in addition, >3000 Arabidopsis membrane proteins in a separate screen are included
PPIM	http://comp-sysbio.org/ppim/	Contains predictions and information form literature
PRIN	http://bis.zju.edu.cn/prin/	Predictions based on interlogs in various model organisms, where studies have been carried out

### Subcellular localization

To predict subcellular localization, and, thus, the third GO domain ‘cellular component’, the general tool TargetP [57] or the secretory signal peptides predictor SignalP [58] are frequently used. These, however, tend to perform poorly in the case of plants [59, 60], so other plant-specific tools such as Plant-mPLoc [61], AtSubP [60] or, for N-terminal targeting sequences, the tool Predotar [62] may produce superior results (Table 2).

However, finding an adequate performance evaluation is often difficult. To avoid biased results, one needs to validate the predictions on a data set, which was not used for training of the predictors [63], and it might be advisable to rely on several tools as is done in the curated reference database for *A. thaliana* protein localization SUBA3 [64].

### Posttranslation modifications

In the case where one is interested in signaling, one can predict phosphorylation sites using four plant tools at the moment, namely PHOSFER [65], PhosPhAt [66], PlantPhos [67] and Musite [68]. In terms of performance, the latter three tools have recently been compared, and it seemed that for serine/threonine predictions, at least in the model *A. thaliana*, Musite performed best. It was, however, noted that for tyrosine phosphorylation, the sensitivity can be lower for Musite at certain specificity ranges [69] (Table 2).

### Predicting function based on expression behavior

Finally, one might venture into functional prediction using nonsequence-based data. A prime example is the ‘guilt by association’ approach, whereby one assumes that a gene to be annotated might exert function X (‘guilt’) if it is co-expressed (‘associated’) with one or several genes of the same known function X [70–73]. The underlying idea is that if several genes consistently show the same expression, there is a good chance that they are co-regulated, as they are needed for the same process or pathway. Insightful examples are macromolecular complexes such as ribosomes, or the cellulose synthesis complex where this guilt-by-association approach works well [74]. Although this approach usually requires many transcriptomic data sets, tissue-specific data sets are often available in genome and/or transcriptome projects, which might prove to be sufficient. Indeed, tissue-specific data might even be helpful to unravel tissue-specific processes, as has been done for *A. thaliana* seed coat mucilage [75, 76]. In the case where metabolic data are available, this might be used to complement the guilt-by-association approach using protocols described recently [77, 78].

Caution needs to be taken, as the guilt-by-association principle is not always reliable and must be evaluated critically. The approach depends on the number of reliably annotated genes within a network. Indeed, even though it works well in cases where queries are restricted to cases similar to the ones listed above (few genes in a well-annotated network), the usefulness of the method decreases when the procedure is scaled up [79].

### Protein interaction databases

A similar approach can be followed using the gene product, the protein, where one searches for interacting proteins. The data repositories of TAIR (Arabidopsis.org) contain such an approach, as well as links to a number of protein interaction resources such as the Arabidopsis interactions viewer [80], IntAct [81], AtPIN [82] and ANAP [83] (Table 2). In addition, GabiPD [84] and PhosPhAt [66] databases hold some information on kinases and their phosphorylation targets, while in the case of membrane proteins, the Membrane-based Interactome Database (M.I.N.D) [85] can be used. The latter is particularly interesting because unlike databases containing various types of curated or predicted data M.I.N.D. rely on experimental results from several rounds of testing using the split-ubiquitin system. The effort for establishing interaction databases is moving onto other plant species, as can be seen in the Protein–Protein Interaction network for Maize (PPIM) [86] and a predicted rice interactome network (PRIN) [87]. A combination of co-expression and interaction data can improve the reliability of the functional prediction [88].

### microRNA and target predictions

In addition to protein-coding genes, other prominent genomic features that regulate gene expressions include noncoding RNAs. This includes a diverse set of plant RNA molecules reviewed in [89, 90], which are transcribed, but never translated into proteins. Next-generation sequencing of these RNA species, which is typically performed using specialized RNASeq libraries targeting small RNAs, has necessitated the development of tools, which can quickly and easily deal with these data sets. An in-depth discussion of all small RNA (sRNA) tools would itself warrant a complete review, so for the purposes of this article, we will restrict our discussion to tools relevant to detection and analysis of microRNA (miRNA) with reference to sRNAtoolbox [91], which offers a selection of user-friendly tools from expression profiling to target gene prediction.

miRNAs are a class of RNA that are involved in gene regulation. Though similar in many respects to small interfering RNA, miRNA can have many target mRNAs and acts as a gene regulator (inhibitor) rather than in gene silencing. To disambiguate the two, guidelines for the annotation of plant miRNAs have been proposed by [92].

One of the main repositories of knowledge for miRNA is miRBase [93]. The most recent release of the database contains 28 645 entries representing precursor miRNAs, expressing 35 828 mature miRNA products, in 223 species. miRBase additionally serves as a registry for newly discovered miRNAs and provides a naming service for miRNA genes. Aside from providing annotations and references for all published miRNA, a ‘Target’ pipeline is provided to predict the targets. However, as this is only aimed at animal miRNA, plant researchers are best referred to a recent benchmark [94] comparing many different plant pipelines. Unfortunately, the outcome was that for species other than *A. thaliana*, the accuracy was generally not too high. It was, therefore, suggested [94] to use a union of predictions stemming from Targetfinder [95] and psRNATarget [96] to maximize finding potential targets at the cost of identifying many false targets. Alternatively, highly confident predictions at the cost of losing many true targets were possible by only using those predictions made by both psRNATarget and Tapir in hybrid mode [97].

## Automated functional annotation pipelines

Given the dramatic increase in genome and transcriptome sequencing, it is not surprising that the demand has grown for fast automated annotation pipelines that quickly provide meaningful biological data from these data sets. Many of the early large-scale genome projects had specific annotation groups assigned to carry out this task, e.g. TIGR for *A. thaliana* [2] and ITAG for *Solanum lycopersicum* [98]. These frequently featured a combination of computational or automated annotations coupled with manual curations. Several recent genome projects have to a greater extent used automated pipelines, which may reflect the increasing quality of the tools available. It should of course be noted that many of the automated pipelines incorporate data, which was manually curated in many of the earlier genomes. Taken together, this also shows that plant genome analysis benefits from the time gain offered by automated tools and increases the focus on analyses of more and different data sets.

In general, these tools can be partitioned based on the underlying ontology used. Probably, the best known tool to infer GO annotations is BLAST2GO [99], which can also incorporate InterProScan and KEGG data. BLAST2GO provides a user-friendly and well-integrated interface featuring locally installed software offering graphical outputs, maps, etc. However, some of these features are not available in the free and academic version but require a license. An alternative, which is aimed at plant researchers who would like to apply GO terms, is provided by the fully integrated TRAPID plant-specific pipeline. TRAPID offers a Web-based analysis platform and alleviates the need to install software [35]. As TRAPID also uses gene families, it usually should provide good annotation performance.

To apply the KO entries (or K numbers) from the KEGG database to a gene set, the popular online tool KEGG Automatic Annotation Server (KAAS) [100] provides a user-friendly interface. This service relies on BLAST searches and either on unidirectional hits or on bidirectional hits together with some heuristics. Recent updates to KEGG have introduced the BlastKOALA and GhostKOALA online tools, which allow users to exploit data from KEGG’s internal annotation tool (KOALA) [101]. Both tools target the nonredundant pangenomic data set generated from KEGG’s genes database, with GhostKOALA using the GHOSTX search algorithm, which is considered more appropriate for metagenome annotation. The result from these tools can be used as input for the other KEGG modules (e.g. KEGG pathways).

The MapMan ontology can be inferred using the online tool Mercator [102]. This allows annotation of both protein and DNA sequences and incorporates BLAST and CDD searches as well as an optional InterProScan annotation. In the case of DNA sequence submission, the file is simply analyzed in all six frames for domain searches, and the annotations merged with the expectation that the correct frame will return the best result. Users can optionally choose a selection of well-annotated plant genomes to be included in the analysis.

### Performing annotations using locally installable resources

For the more computation savvy researcher who has access to decent computing resources, Trinotate (https://trinotate.github.io) offers a comprehensive annotation suite, which extends the popular Trinity RNASeq assembly pipeline [8]. It comprises a BLAST search against the manually assigned SWISSPROT data set, HMMER searches against the PFAM database as well as finding signal sequences and predicting subcellular localization using TMHMM [53] and SignalP [58], respectively. In addition, it allows inclusion of the RNAmmer [103] tool which is used to identify rRNA transcripts and integrates a selection of annotation databases (KEGG, EggNOG and GO). An alternative is the plant-specific AHRD pipeline (https://github.com/groupschoof/AHRD), which provides a consensus annotation based on a set of input gene descriptions obtained by sequence similarity searches. The annotations are the result of scoring the input gene annotations according to both their frequency and the reputation of the database from which it was derived.

The sets of tools described in the previous two sections using controlled terms or annotations are often preferred, given the ease with which the results can be compared against other similarly annotated genomes. Prominent examples that use these tools include the melon genome, which used KAAS [104], the peanut ancestor genome, which used AHRD [105], and the genome of the wild tomato, which used Mercator [106].

### An example of annotation pipelines using rapeseed proteins

Table 3 provides a small survey of the integrated tools by annotating 1476 rapeseed proteins using the automated annotation pipelines. It is evident that the tools relying on their own infrastructure generally deliver results quickly. In addition, the annotation rate ranges from 26% for the KEGG-based tools, likely based on KEGG’s stronger focus on metabolism, to 56% for GO or MAPMAN-based terms. The latter value compares with the annotation rate of about 51% (from the downloaded reference) when counting any GO term (including ‘cellular component’). In contrast, BLAST2GO reaches a higher annotation rate of 78% but requires 10× more time when run on a typical workstation type laptop (e.g. i7 Quadcore). As noted above, such a high annotation rate (especially as it is higher than the reference) could result from aggressive (and thus sensitive) standard settings, which potentially should be further tuned when annotating plant genomes. Nevertheless, BLAST2GO might provide valuable leads into less likely functions, and as was the case for TAIR, most annotations were for the ‘Cellular Component’ domain, as for 70% of the genes a cellular component domain GO term could be determined. It is noteworthy that both plant-specific pipelines (TRAPID and Mercator) reach similar annotation rates, which is likely because of their specific fine tuning to plant-derived proteins.

**Table 3.** Integrated tools for the functional analysis of plant genomes

Resource	Time taken	Annotation rate (%)	Comments
Reference	—	51	At least one GO term assigned including cellular component
Blast2GO	8 h 23 min	78	BLAST is performed locally or as WebBLAST via NCBI; InterProScan is performed as a Web service at the European Bioinformatics Institute (EBI)
KAAS	10 min (only single- directional best hit (SBH) was used as a survey sample of sequence)	29	Runs as a Web service, no user resources needed
GhostKOALA	28 min	26	Runs as a Web service, no user resources needed
Mercator	5 min	56	Runs as a Web service, no user resources needed
TRAPID	5 min	56	Runs as a Web service, no user resources needed

*Note*. For the analysis, the first 1476 proteins from the Brassica proteome version 5 were downloaded from http://www.genoscope.cns.fr/brassicanapus/data/ alongside their GO annotations, representing exactly 10 000 lines of text and submitted to the various services, where available searches were limited to plant data sets. In the case of Blast2GO, WebBLAST was used. We have rounded the values, as annotations are subjected to updates, and time taken will depend on server loads. Therefore, these values should be seen as a general orientation.

Integrating the output from several pipelines has been shown to be beneficial, such as in the case of the potato crop [107]. In this pipeline, the authors used Trinotate, BLAST2GO, OrthoMCL together with other tools to produce an Ensemble classifier by counting how many different pipelines a certain GO term was detected. Interestingly, by using even simple Ensembles, they increased the concordance with literature annotations. A similar multiple site data retrieval strategy was used for the wheat database dbWFA [108], which provides a data warehouse strategy and, thus, allows querying and combining different annotations per wheat gene to provide a more comprehensive picture.

## Potential pitfalls and difficult gene families

Generally speaking, the high-throughput tools mentioned above use methods, which can quickly identify the general gene function. This frequently relies on identifying the protein family based on the aforementioned HMMs. Although this is often sufficient, there are many cases where a finer-grained approach is necessary. Plant genomes are renowned for containing large gene families such as transcription factors, which can easily number in the thousands. The Cytochrome P450 family of genes is known to be large in many organisms, and it is frequently the target of scientific interest in plants given their prominent role in secondary metabolite biosynthesis, which is particularly important to medicinal plants. Indeed, the two large projects PhytoMetaSyn [109] and Medicinal Plant Genome Resource [110] are dedicated to the transcriptome analysis of medicinal plants.

In cases of large or difficult gene families, it is often necessary to analyze data in detail by building gene family trees, which first require careful multiple sequence alignments. This dismantles large groups of genes into individual genes or smaller clades allowing distinct functions to be applied. An alternative and/or complementary approach could involve analyzing syntenic relationships using the CoGE resource [111]. There are many resources available that are dedicated to annotating difficult gene families such as PlantTFDB and Potsdam PlnTFDB for plant transcription factors [112–114], the P450 database for P450 enzymes [115], CAZy for carbohydrate active enzymes [116], Merops for peptidases [117] and Aramemnon for plant membrane proteins [55], which are summarized in Table 4. These are complemented by the more generalist plant family database PLAZA [15] and GreenPhylDB [118]. On a broader level, using detailed phylogenetic information in the genomic era brings in phylogenomics tools whose use is reviewed in [119].

**Table 4.** Tools and Web sites useful in annotating large protein families

Resource	Function	Web address
CoGe	Compares genomes, find synteny	https://genomevolution.org
PlantTFDB	Plant Transcription Factor families	http://planttfdb.cbi.pku.edu.cn/
Potsdam plntfdb	Plant Transcription Factor families	http://plntfdb.bio.uni-potsdam.de/v3.0/
P450 Database	P450 protein families	http://drnelson.uthsc.edu/CytochromeP450.html
CAZy	Enzymes acting on carbohydrates	http://www.cazy.org/
Aramemnon^a^	Plant membrane proteins	http://aramemnon.uni-koeln.de/
Merops Database	Peptidases	http://merops.sanger.ac.uk
PLAZA	Generalist Plant Family database	http://bioinformatics.psb.ugent.be/plaza
GreenPhylDB	Generalist Plant Family database	www.greenphyl.org/

*Note*. ^a^Also lists a comprehensive set of tools for transmembrane domains, subcellular localization and lipid modifications.

## Recipe

**Figure 2.**
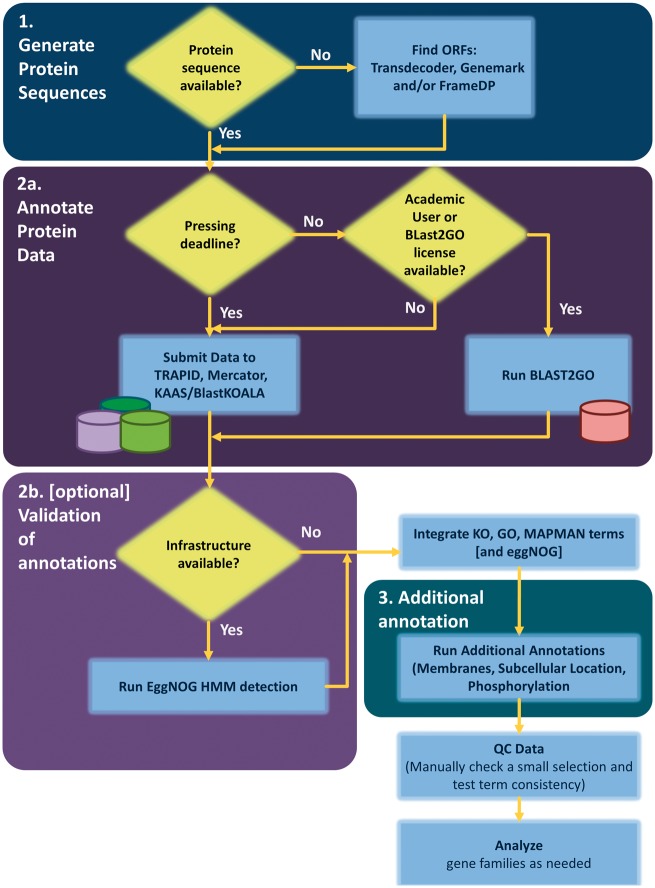
Flowchart for the annotation of plant genomes/transcriptomes.

Based on the discussion above and focusing on the use of online resources, one could annotate a plant genome almost automatically following the steps below (Figure 2):

(i) Generation of protein sequences: The first question one should ask is whether protein sequences are available (which is typically the case in a genome project) or not (which is typically the case in a transcriptome project). If protein-coding sequences are not available, these should be generated from transcript sequences using, e.g., FrameDP or the AUGUSTUS/BREAKER1 pipeline for genome assemblies. Other tools to perform this task are discussed in the section ‘Finding coding regions in transcriptome assemblies’. This would provide a common input for the subsequent annotation regardless of the starting approach.

(iia) Annotation: One would then submit the resulting protein sequences as one file to the following three online resources. As all these services make use of their own high-performance computing pipelines and are free for academic users, they can be run in parallel.

- KAAS can be used to infer KEGG terms.
- The TRAPID pipeline can be used for GO terms.
- Mercator can be used for MapMan terms.

At this stage, one could also use BLAST2GO to infer GO annotations; however, this is only possible if one either has a license or is an academic user. It should be considered, however, that BLAST2GO has a much longer run-time, which could impede subsequent genome annotation analysis tasks. Thus, a decision is needed if the additional time is worth the extra annotations, which are potentially not provided by annotation alternatives like TRAPID.

(iib) (optional) Validation of annotations: In cases where local computational resources are available, one should additionally run EggNOG scans on the side, to further validate and compare the derived functional annotations. To test if this procedure is feasible using the available equipment, we recommend running a truncated sample of, e.g., 100–1000 protein sequences first.

The above two steps provide a fast solution to arrive at a plethora of terms, which can be easily combined using even simple tools like MS Excel, where one could add the different ontologies into separate rows for inspection. Even though the different ontologies cannot be directly compared with each other, they help in understanding the genome in their own right.

This would already provide a good working annotation for many research topics and could be used to answer questions such as Are certain processes occurring or not? Or do we see more genes in secondary metabolism than in related plants?

(iii) Additional information: One can annotate transmembrane domains using TMHMM and/or TOPCons (the online versions of both were relatively easy to use in our hands, but the online TMHMM tool was significantly faster). For TMHMM, one might have to split the protein sequence file obtained from Step (i) into several batches. In the simplest case, one could do this by hand in a text editor such as Notepad ++ on Windows or TextWrangler on MacOS.

(iv) Similarly, as for transmembrane domains (i.e. after splitting of the file from Step (i)), subcellular localization can be predicted using the online tools TargetP and/or AtSubP.

(v) Finally, one could use the PhosPhat and PHOSFER online tools for a prediction of phosphorylation sites.

After the annotation process is complete, it is advisable to look at a selection of these annotations to verify whether they are correct. A good choice for a more experiment-oriented researcher would be to focus on the genes or gene families, which one works with in the laboratory. This verifies that the expected annotations are present and that no wrong annotations had been added. Alternatively, or in addition, manually comparing genes described in the literature with their automatically derived annotations are highly recommended.

Key Points

- Annotating plant genomes should (also) rely on ontologies, and there are several complementary resources available.
- Generally, large-scale annotation relies on homology transfer, and it is complemented by finding domains and protein families.
- Annotation can be further complemented by additional predictions such as transmembrane domains, subcellular localization and phosphorylation sites.
- Plant genome annotation can be performed automatically using the plant-specific tool Mercator as well as the generalists TRAPID (which features a special plant module), KAAS/BlastKOALA and Blast2GO without any significant computational resources.
- It is important to quality check the annotation and to remember that all annotations should be treated as hypotheses.

## Funding

The German Ministry for Education and Research (reference number 0315961, in partial), the Ministry of Innovation, Science and Research of North-Rhine Westphalia within the framework of the North-Rhine Westphalia Strategieprojekt BioEconomy Science Center (grant number 313/323–400–00213) and the F.R.S-FNRS Fonds de la Recherche Scientifique, as a Chargé de recherches (grant number 10841946).

## Notes

**Marie Bolger** is a postdoctoral fellow at the Forschungszentrum Jülich (Jülich, Germany) working on the German Plant Primary Database.

## Notes

**Borjana Arsova** is an FNRS Researcher at the Department of Life Sciences, University of Liège, (Liège, Belgium), and currently a guest scientist at IBG-2 Plant Sciences at the Forschungszentrum Jülich (Jülich, Germany). She uses proteomics for the study of nutrient uptake in plants.

## Notes

**Björn Usadel** is a professor at the RWTH Aachen University (Aachen, Germany) and Director at the IBG-2 Plant Sciences at the Forschungszentrum Jülich (Jülich, Germany) where he leads the bioinformatics group. He is also core group leader at the BioEconomy Science Center BioSC.de, where he is mostly investigating plant gene function and cell wall decomposition.
